# Discordant American Society of Anesthesiologists Physical Status Classification between anesthesiologists and surgeons and its correlation with adverse patient outcomes

**DOI:** 10.1038/s41598-022-10736-5

**Published:** 2022-05-02

**Authors:** Charlene Xian Wen Kwa, Jiaqian Cui, Daniel Yan Zheng Lim, Yilin Eileen Sim, Yuhe Ke, Hairil Rizal Abdullah

**Affiliations:** 1 Department of Anaesthesiology, Singapore General Hospital, Outram Road, Singapore, 169608 Singapore; 2 Health Service Research Unit, Medical Board, Singapore General Hospital, Outram Road, Singapore, 169608 Singapore; 3 Duke-NUS Medical School, 8 College Road, Singapore, 169857 Singapore

**Keywords:** Diseases, Medical research, Health care, Health care economics, Health policy, Health services

## Abstract

The American Society of Anesthesiologists Physical Status Classification (ASA) is used for communication of patient health status, risk scoring, benchmarking and financial claims. Prior studies using hypothetical scenarios have shown poor concordance of ASA classification among healthcare providers. There is a paucity of studies using clinical data, and of clinical factors or patient outcomes associated with discordant classification. The study aims to assess ASA classification concordance between surgeons and anesthesiologists, factors surrounding discordance and its impact on patient outcomes. This retrospective cohort study was conducted in a tertiary medical center on 46,284 consecutive patients undergoing elective surgery between January 2017 and December 2019. The ASA class showed moderate concordance (weighted Cohen’s κ 0.53) between surgeons and anesthesiologists. We found significant associations between discordant classification and patient comorbidities, age and race. Patients with discordant classification had a higher risk of 30-day mortality (odds ratio (OR) 2.00, 95% confidence interval (CI) = 1.52–2.62, *p* < 0.0001), 1-year mortality (OR 1.53, 95% CI = 1.38–1.69, *p* < 0.0001), and Intensive Care Unit admission > 24 h (OR 1.69, 95% CI = 1.47–1.94, *p* < 0.0001). Hence, there is a need for improved standardization of ASA scoring and cross-specialty review in ASA-discordant cases.

## Introduction

The American Society of Anesthesiologists physical status classification (ASA class) is a widely utilized grading system first introduced in 1941^1^ and revised in 1961^2^ to assess and communicate the preoperative health of patients undergoing anesthesia. It consists of six categories ranging from Class 1 (describing a healthy patient) to Class 6 (referring to the brain-dead organ donor). Clinical examples for each ASA class were added in 2014^3^ with the aim of improving inter-rater reliability or concordance^4^.

ASA scoring has significance both clinically and from a health services perspective. While ASA scoring alone is not intended for the prediction of perioperative risks^5^, it has been shown to be independently predictive of perioperative morbidity and mortality^6^ and is included as part of several perioperative risk assessment tools that are widely used by surgeons and anesthesiologists. These include the National Surgical Quality Improvement Program risk calculator^7^, Gupta Myocardial Infarction or Cardiac Arrest calculator^8^, Surgical Outcome Risk Tool^9^ and Combined Assessment of Risk Encountered in Surgery^10,11^. Discordance in ASA classification between healthcare providers is therefore concerning and may subject patients to contradictory risk counseling and inappropriate perioperative plans. At a health system level, discordant ASA scoring may undermine efforts for quality assurance^12^, allocations of critical care resources, risk-based remuneration for health outcomes and may result in potential financial costs from over-scoring^13^. ASA classes are also frequently reported in healthcare benchmarking exercises and payer billing documentations.

Multiple studies have reported moderate to poor concordance of the ASA class among various clinicians^14^, anesthesiologists^15–20^ or restricted to specific patient cohorts^21–23^. One study examined specifically the agreement between anesthesiologists and surgeons using hypothetical patient scenarios^24^. There is a paucity of clinical data in this particular area. This is an important evidentiary gap as both specialties jointly manage patients undergoing surgeries. Furthermore, the association between discordant ASA classification and adverse patient outcomes has not been comprehensively studied previously.

To fill these knowledge gaps, our study aims to examine the concordance of ASA classification between surgeons scheduling patients for surgery and anesthesiologists conducting the outpatient preoperative evaluation. We further examined the clinical and demographic factors associated with discordant classification and whether discordant classification was associated with adverse postoperative outcomes.

## Methods

### Study design and data sources

This was a single-center retrospective cohort study conducted in Singapore General Hospital, the largest tertiary academic medical center in Singapore. It is a Level 1 Trauma Center and has all major surgical specialties other than pediatric surgery. The Singhealth Centralized Institutional Review Board (CIRB Reference Number 2020/2801) granted a waiver of consent due to the use of anonymized routinely collected clinical data and no patient interaction was required. All experimental protocols were approved by the Singhealth Centralized Institutional Review Board. The data analysis and statistical plan was written and filed before the data were accessed. All methods were carried out in accordance with relevant guidelines and regulations.

Our study cohort was extracted from the Perioperative and Anesthesia Subject Area, a curated electronic medical records database within our institution’s enterprise data warehouse (SingHealth-IHiS Electronic Health Intelligence System) which contains the records of all operative procedures performed since 2015. The system integrates patient information such as patient demographics, laboratory results, comorbidities and postoperative outcomes from multiple healthcare transactional systems, such as the hospital’s clinical information system (Sunrise Clinical Manager, Allscripts, Illinois, United States of America) and other administration and ancillary electronic systems. Mortality data on the system were synchronized with the National Electronic Health Records, including data from the National Registry of Births and Deaths, ensuring a near-complete mortality data follow-up.

In our institution, the ASA class is assigned by the surgeon on a standardized electronic admission form during the surgery listing process. Patients are then typically seen in the anesthesia preoperative clinic within a month of the surgery listing. Information on patient demographics, anthropometric parameters, preoperative comorbidities, and ASA class are routinely assessed by the attending anesthesiologist as part of structured clinical notes during the preoperative assessment, and are included within the database. The 2014 ASA scoring definition along with their published examples are available for reference in the anesthesia preoperative clinic and surgery clinic and is attached in Supplementary table S1. The ASA classification by both anesthesiologists and surgeons in this study is hence based on the 2014 revision. While the anesthesiologist can potentially access the surgeon’s ASA class, it is usually independently assigned in our center. There are no financial incentives in assigning a higher ASA class both for anesthesiologists and surgeons within our local healthcare system.

### Participant cohort and variables

**Figure 1:**
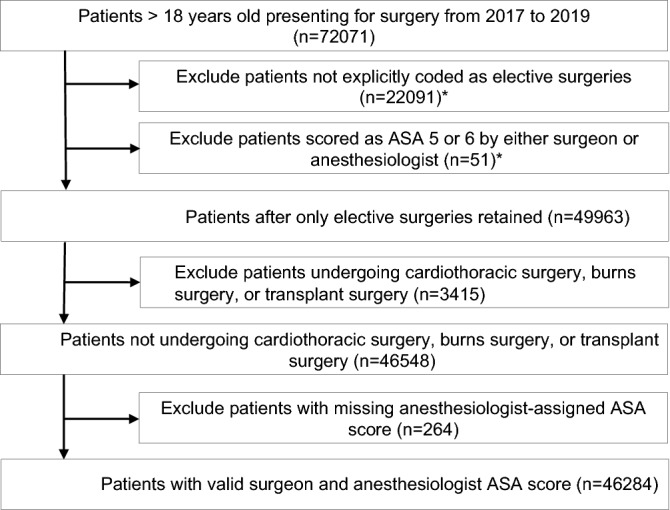
Study flow diagram for patient cohort definition. *The exclusions for patients not explicitly coded as elective surgeries and patients with ASA 5 or 6 are overlapping categories, and as a result sum to more than the difference between the first two steps.

We included all patients aged 18 years old and above undergoing elective surgery under general or regional anesthesia or monitored anesthesia care between January 2017 and December 2019. Patients who underwent cardiothoracic surgery, transplant surgery, or surgery for burns injuries were excluded. Patients planned for elective cardiothoracic surgery in our center have the ASA field in the preoperative structured clinical note filled by the surgeon themselves (unlike other surgical patients, where the ASA field would be populated by an anesthesiologist), while patients requiring transplant surgery would usually have a standardized ASA class as there is organ failure necessitating the surgery. Patients with a missing ASA class by either the surgeon or anesthesiologist and patients assigned an ASA class of 5 or 6 by either the surgeon or anesthesiologist were also excluded (Fig. 1).

For each patient, we obtained preoperative data such as age, sex, race, surgical specialty, and comorbidities including ischemic heart disease, congestive heart failure, cerebrovascular accidents, diabetes mellitus requiring insulin, and hypertension. These comorbidities are assessed by the anesthesiologist as part of the Revised Cardiac Risk Index, which is routinely used in our institution^25^. The ASA classes assigned by both the anesthesiologist and surgeon were obtained, and the relevant clinical outcomes (death within 30 days, death within 1 year, ICU admission for > 24 h) were determined.

Supplementary table S2 compares the characteristics of 264 patients who were excluded from our study as they had no valid ASA class. All 264 patients had missing anesthesiologist ASA class and there was no statistically significant difference between patients in the final cohort and the excluded patients for demographic variables (age, sex, race) and clinical outcomes. Among the excluded patients, there were fewer that had anesthesiologist-assessed comorbidities, and the differences were statistically significant for some. Our interpretation is that patients with incomplete anesthesiologist ASA class were more likely to have other areas incompletely assessed by the anesthesiologist. Overall, the number of such patients is small and not deemed to be a major source of bias.

### Statistical analysis

Analyses were performed using Python version 3.7.1 and R version 4.0.2 with their base utility functions. Additional packages used in R included the “questionr” package for logistic regression, “pROC” for receiver operating characteristic curve analyses, and “irr” for concordance analyses.

#### Assessment of agreement between surgeon and anesthesiologist ASA classification

Cross tabulation was performed for the anesthesiologist’s ASA class against the surgeon’s ASA class. Concordance between these two variables was determined using Cohen’s weighted κ. The κ-statistic was interpreted in the manner of Altman as poor (0–0.2), fair (0.21–0.4), moderate (0.41–0.6), good (0.61–0.8) and very good (0.81–1.0) agreement^26^.

Our sample was drawn from a database that exhaustively documents all surgeries performed within the hospital, and we considered all sequential patients within the study time frame (January 2017–December 2019). As a comparison, the sample size calculation to detect a moderate agreement (κ > 0.4) and exclude a fair agreement (κ = 0.2) with a one-sided 95% confidence interval and 90% power is 186.

#### Descriptive statistics for overall cohort and subgroup analyses of discordant ASA classes

Descriptive statistics were calculated and expressed as counts and percentages for categorical data, and means with standard deviation for continuous data. The cohort was stratified into patients with concordant and discordant ASA classes. Univariate statistical analysis was performed using the chi-square test for categorical variables and the t-test for continuous variables. Subgroup analyses were also performed comparing patients where the surgeon assigned a lower ASA class against patients with a concordant ASA class, and likewise comparing patients where the anesthesiologist assigned a lower ASA class against patients with a concordant ASA class. In view of the multiple statistical comparisons, Bonferroni’s correction was used and the p-value cut-off for statistical significance was determined to be *p* < 0.001.

#### Effect of discordant ASA classification on clinical outcomes

The discordance of ASA classification between surgeons and anesthesiologists was calculated and stratified in several different ways. Three forms to express discordance were used. Firstly, as a binary variable representing whether the ASA classes were discordant or not; secondly, as a ternary variable representing whether the ASA classes were concordant, surgeon ASA class was lower, or anesthesiologist ASA class was lower; and lastly, as the raw difference with appropriate binning of categories with low counts. These variables, representing different ways of stratifying the degree of ASA classification discordance, were separately entered as the sole predictive variable into logistic regression models. A separate model was fitted for each of the clinical outcomes of death within 30 days, death within 1 year, and ICU admission for > 24 h. The unadjusted odds ratios and p-values were calculated for each stratum of ASA discordance, with the ASA concordant patients as the reference group.

In this analysis, we did not include any predictive factors besides ASA discordance. This is because ASA discordance should in theory have no significant effects on clinical outcome and it cannot be regarded as a prognostic marker per se. Rather, any significant effect of ASA discordance would suggest that it is a red flag indicator of potential shortcomings in the clinical care process. Any underlying factor to the ASA discordance, would necessarily have a collinear relationship with the ASA discordance itself. Hence it would not be appropriate to enter other factors alongside ASA discordance into the same regression model, particularly if these factors are suspected to be the cause of ASA discordance itself. These are analyzed separately in the prior section.

## Results

### Concordance of surgeon and anesthesiologist ASA classification

Our final study cohort comprised 46,284 patients, of which 46.4% (21,474/46,284) were male and 53.6% (24,810/46,284) were female. The cross-tabulation of surgeon and anesthesiologist ASA class for all cases is presented in Table 1. The weighted Cohen’s κ for concordance between surgeon and anesthesiologist class was 0.53, signifying moderate agreement.

**Table 1:** Cross-tabulation of ASA classes by surgeon and anesthesiologist.

		ASA classified by surgeon			
		1	2	3	4
**ASA classified by anesthesiologist**	1	4247	2160	7	0
	2	*5014*	22,996	867	8
	3	285	*6364*	3869	71
	4	1	86	235	74

The ASA classification by both surgeon and anesthesiologist for each patient is presented. Italicized values demonstrate the two greatest areas of discordance ASA 2 by anesthesiologist and ASA 1 by surgeon (5014 patients), as well as ASA 3 by anesthesiologist and ASA 2 by surgeon (6364 patients).

### Descriptive statistics and stratified analyses

67.4% of patients (31,186/46,284) had a concordant ASA class given by both surgeons and anesthesiologists. Descriptive statistics for the ASA concordant and discordant groups are presented in Table 2. 79.4% of patients with discordant classes (11,985/15,098) had a lower ASA class assigned by the surgeon, and 20.6% (3113/15,098) had a lower ASA class assigned by the anesthesiologist.

**Table 2:** Baseline patient characteristics stratified by the concordance of ASA classes given by surgeons and anesthesiologists.

	Overall (n = 46,284)	Concordant ASA class (n = 31,186)	Discordant ASA class (n = 15,098)	*p* value^1^	Surgeon ASA lower (n = 11,985)	*p* value^2^	Anesthesiologist ASA class lower (n = 3113)	*p* value^3^
Male sex, no. (%)	21,474 (46.4)	14,312 (46.0)	7162 (47.4)	0.0002	5670 (47.3)	0.0072	1492 (47.9)	0.031
Age (time of surgery), mean (SD)	58.0 (16.0)	59.0 (16.0)	56.0 (17.0)	< 0.0001	56.0 (17.0)	< 0.0001	54.0 (16.0)	< 0.0001
**Race, no. (%)**								
Chinese	34,560 (74.7)	23,814 (76.4)	10,746 (71.2)	< 0.0001	8463 (70.6)	< 0.0001	2283 (73.3)	< 0.0001
Indian	4459 (9.6)	2793 (9.0)	1666 (11.0)		1336 (11.2)		330 (10.6)	
Malay	4111 (8.9)	2558 (8.2)	1553 (10.3)		1306 (10.9)		247 (7.9)	
Others	3154 (6.8)	2021 (6.5)	1133 (7.5)		880 (7.3)		253 (8.1)	
Creatinine > 2 mg/dl, no. (%)	2281 (4.9)	1507 (4.8)	774 (5.1)	0.18	722 (6.0)	< 0.0001	52 (1.7)	< 0.0001
Diabetes mellitus on Insulin, no. (%)	1750 (3.8)	1025 (3.3)	725 (4.8)	< 0.0001	681 (5.7)	< 0.0001	44 (1.4)	< 0.0001
History of Congestive heart failure, no. (%)	1065 (2.3)	608 (2.0)	457 (3.0)	< 0.0001	432 (3.6)	< 0.0001	25 (0.8)	< 0.0001
History of Cerebrovascular accident, no. (%)	1529 (3.3)	799 (2.7)	730 (4.8)	< 0.0001	683 (5.7)	< 0.0001	47 (1.5)	0.00039
History of Ischemic heart disease, no. (%)	4635 (10.0)	2500 (8.0)	2135 (14.1)	< 0.0001	1970 (16.4)	< 0.0001	165 (5.3)	< 0.0001
History of Hypertension, no. (%)	19,225 (41.5)	13,591 (43.6)	5634 (37.3)	< 0.0001	5070 (42.3)	0.021	564 (18.1)	< 0.0001
History of Smoking, no. (%)	4327 (9.4)	2672 (8.6)	1655 (11.0)	< 0.0001	1434 (12.0)	< 0.0001	221 (7.1)	0.0055
**Surgical specialty, no. (%)**								
Orthopedics	14,523 (31.4)	10,472 (33.6)	4051 (26.8)	< 0.0001	2958 (24.7)	< 0.0001	1093 (35.1)	< 0.0001
General surgery	11,294 (24.4)	7501 (24.1)	3793 (25.1)		3062 (25.6)		731 (23.5)	
Urology	6403 (13.8)	4472 (14.3)	1931 (12.8)		1503 (12.5)		428 (13.8)	
Obstetrics and gynecology	4799 (10.4)	2803 (9.0)	1996 (13.2)		1826 (15.2)		170 (5.7)	
Otorhinolaryngology	2844 (6.1)	1817 (5.8)	1027 (6.8)		876 (7.3)		151 (4.9)	
Vascular	2146 (4.6)	1418 (4.6)	728 (4.8)		582 (4.9)		146 (4.7)	
Plastics	1468 (3.2)	942 (3.0)	526 (3.5)		388 (3.2)		138 (4.4)	
Neurosurgery	730 (1.6)	399 (1.3)	331 (2.2)		231 (1.9)		100 (3.2)	
Others	2077 (4.5)	1362 (4.4)	715 (4.7)		559 (4.7)		156 (5.01)	

Descriptive statistics for the ASA concordant and discordant groups. 79.4% of patients with discordant classes (11,985/15,098) had a lower ASA class assigned by the surgeon, and 20.6% (3113/15,098) had a lower ASA class assigned by the anesthesiologist.

^1^*p* value for difference between ASA concordant and discordant patients.

^2^*p* value for difference between ASA concordant patients and those classified lower by the surgeon.

^3^*p*value for difference between ASA concordant patients and those classified lower by the anesthesiologist.

**Figure 2:**
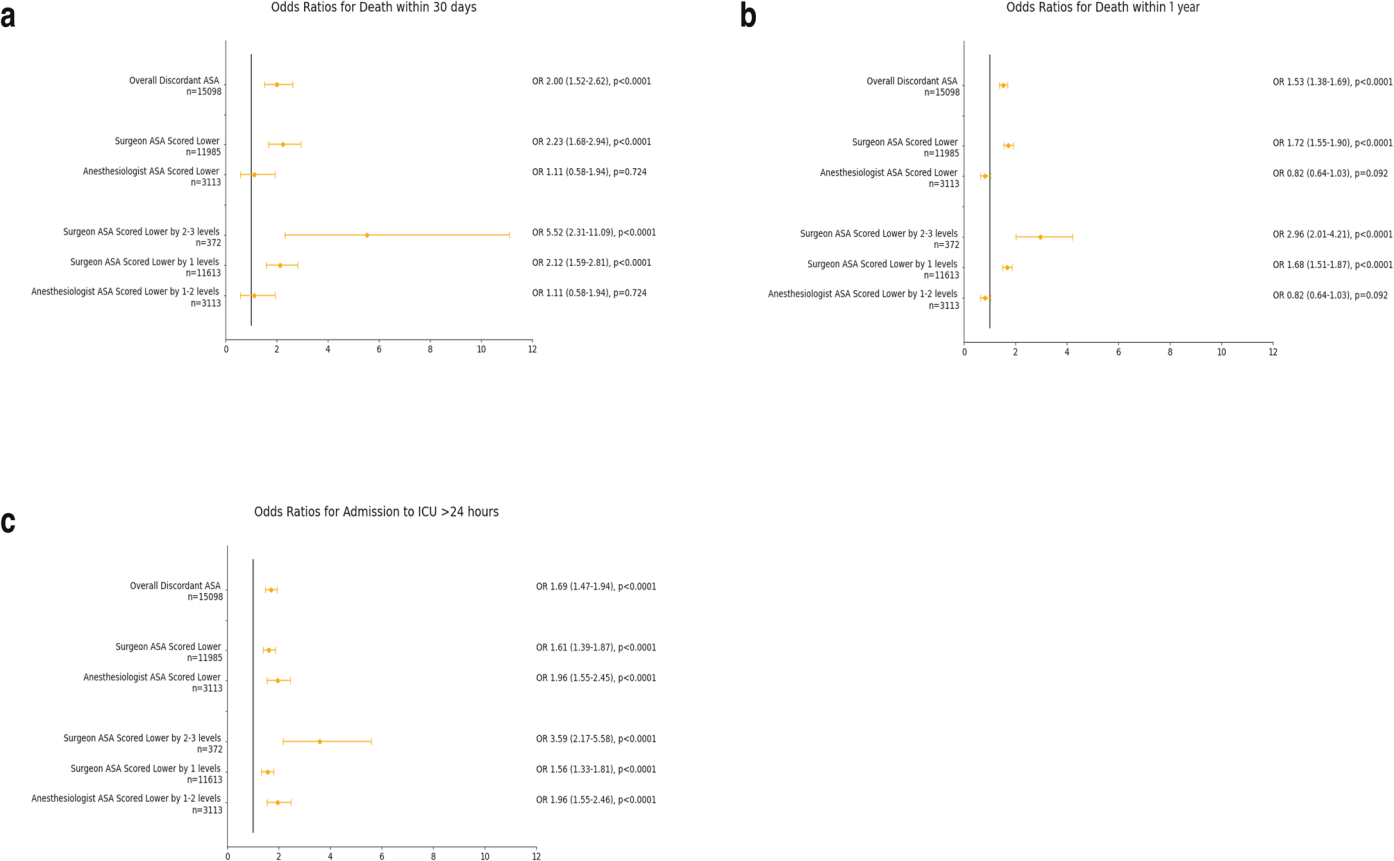
Odds Ratio Plots for Risk of Adverse Outcomes with Different Levels of ASA Discordance. (**a**) Odds Ratio for death within 30 days; (**b**) Odds Ratio for death within 1 year; (**c**) Odds Ratio for ICU admission > 24 h. A lower surgeon ASA class as compared to the anesthesiologist class was associated with all three outcomes. On the other hand, a lower anesthesiologist ASA class was only associated with ICU admission > 24 h but not death at 30 days or 1 year.

For all baseline patient characteristics, there were significant differences in the presence of comorbidities between patients with concordant and discordant classes, with exception of the male sex and the presence of raised creatinine. Comorbidities that were present in a higher proportion of patients with discordant ASA class compared to those with concordant ASA class include a history of ischemic heart disease (14.1% vs. 8%, *p* < 0.0001), cerebrovascular accident (4.8% vs. 2.7%, *p* < 0.0001) and congestive heart failure (3% vs. 2%, *p* < 0.0001) and the presence of diabetes mellitus on insulin (4.8% vs. 3.3%, *p* < 0.0001). Patients with discordant ASA classifications also had a younger mean age (56 vs. 59 years old, *p* < 0.0001). All surgical specialties which were included also had significant differences with respect to discordance. Discordant ASA classification overall was associated with a higher risk of all adverse outcomes- death at 30 days, death at 1 year, and ICU admission of more than 24 h. When the discordant ASA classes were further stratified, we observed that a lower surgeon ASA class was associated with all negative outcomes. For patients where the surgeon ASA class was lower, the risk of a negative outcome was increased when there was greater difference between the surgeon and anesthesiologist ASA classification. On the other hand, a lower anesthesiologist ASA class was only associated with ICU admission > 24 h but not death at 30 days or 1 year. This is depicted in Fig. 2, with additional details included in Supplementary table S3.

We also conducted a subgroup comparison of the effects of discordant ASA class on clinical outcomes within the lower ASA class 1–2 groups, as well as within the higher ASA class 3–4 groups (Supplementary table S4). This showed significant difference in clinical outcomes when the discordance was between ASA class 3 and 4, whereas there was no difference in clinical outcomes when the discordance was between ASA classes 1 and 2.

## Discussion

### General discussion

Our results demonstrate differences in ASA classification between surgeons and anesthesiologists in clinical practice after the addition of clinical examples in 2014, which have previously been studied only in hypothetical scenarios^24,27^ or between anesthesiologists and Internal Medicine providers^14^. Furthermore, we found that discordant ASA classification is associated with adverse outcomes, particularly when the surgeon-assigned ASA class is lower.

The observed moderate concordance (κ 0.53) in our study is consistent with that reported in the retrospective cohort study by Sankar et al. between anesthesiologists in the preoperative clinic and on the day of surgery (κ value 0.61) before the 2014 ASA update^28^. Another study by Abouleish et al. of concordance between anesthesiologists in the preoperative clinic and on the day of surgery had similar results (κ value 0.62), but subsequently demonstrated ‘very good’ agreement (κ value 0.85) after the introduction of examples that were ASA and institutionally approved^29^.

The majority of discordant classification involved a lower class assigned by surgeons, with the largest group comprising those assigned ASA 3 by the anesthesiologist but ASA 2 by the surgeon. We observed that patients with discordant ASA class had a significantly higher proportion of comorbid clinical conditions (raised creatinine, diabetes mellitus on insulin, history of congestive heart failure, cerebrovascular accident, ischemic heart disease and smoking). This reflects the continuing subjectivity of the ASA scoring system despite the 2014 update, which was intended to improve concordance. The differences in recognition and perceived significance of comorbidities are likely to be a major contributing factor to discordant ASA classification. Of note, ASA-approved examples are not present on both the electronic forms used by the surgeon and the anesthesiologist. However, both groups of physicians have been familiarized with the classification and its examples via both regular and ad-hoc training sessions, and a hard copy of the examples are available in the both clinics’ resource folder for convenient perusal. There may be further need for standardization and education efforts in both clinics following this study.

As the ASA class is a component of several major surgical risk scoring systems used by both surgeons and anesthesiologists in clinical care, discordant ASA classification can adversely impact the reliability of perioperative risk scoring and subsequent risk counseling. The ASA class is routinely used in deciding what preoperative tests a patient requires at our institution and in other countries such as the United Kingdom^30^. Overestimation of the ASA class would increase the number of investigations a patient has before surgery, incurring unnecessary financial costs to the patient and healthcare system, while an underestimation of the ASA class may compromise patient safety. At the health system level, discordant classification can also affect the allocation of critical care resources and undermine the use of the ASA class in healthcare reimbursement and quality assurance efforts. This may disadvantage healthcare institutions financially and in inter-institutional rankings depending on which class is being reported to the external agencies. Other studies have shown that the addition of examples to the ASA class and reinforcement of its use were required to improve reliability^4,29^. Standardization efforts are needed to improve the utility of ASA classification in clinical practice and for uses beyond the original intention of communicating patient healthcare status.

We also note that certain demographic factors were associated with discordant ASA classification, such as in younger patients and those of minority ethnicity. We postulate that younger patients may be perceived to have lower severity of disease by some clinicians, hence grading them with a lower class. Minority race patients may face communication or cultural barriers in disease and symptom communication and this may adversely affect accurate healthcare assessment. Additionally, there could be an element of implicit racial bias among healthcare professionals against minority race patients, which has been exhibited in healthcare settings^31^. Ideally, demographic factors should not influence ASA scoring, which should be an objective reflection of patient physical status. This finding further supports the need for better standardization and education on ASA scoring. Further studies on special populations, such as pregnant patients, may also be useful.

Our study revealed that patients with discordant ASA classification had poorer clinical outcomes. All ASA discordant patients had a higher risk of ICU admission > 24 h, in overall and stratified analyses.

With respect to mortality, stratified analyses of discordant ASA classification showed that patients whose surgeon assigned a lower class had a higher risk of 30-day and 1-year mortality. The lower the surgeon ASA class was compared to the anesthesiologist ASA class, the higher the risk was for 30-day and 1-year mortality. In contrast, patients with discordant ASA who were classified lower by their anesthesiologist did not have such an association. This is noteworthy, given that simple differences in medical opinion leading to discordant patient assessments would not ordinarily be expected to correlate with patient outcomes. Failure to recognize a high perioperative risk patient or interval development of comorbidity in the short timespan between surgeon and anesthesiologist review could have contributed to the poorer patient outcomes seen in this group. A breakdown in communication of identified risks between surgeon and anesthesiologist may also be a significant mechanism by which ASA discordance may occur. However, in the absence of independent adjudication, it would be difficult to ascertain the extent to which this applies.

Finally, discrepancy between ASA class 1–2 grading among surgeons and anesthesiologists had no significant correlation with clinical outcomes, whereas discrepancy between the higher classes of 3–4 was significantly associated with death at 30 days and ICU admission > 24 h (Supplementary table S4). Further training should emphasize the importance of distinguishing the higher ASA classes as discrepancy at this level will have a significant impact on clinical outcomes.

### Study strengths and limitations

Our study’s main strengths are that it was conducted in a large patient cohort spanning multiple years and encompassing the major categories of elective noncardiac surgery. Data collected was from 2017 to 2019, after the 2014 ASA update and with adequate time-lapse for familiarization, and before the 2020 ASA update to include clinical examples for obstetric and pediatric patients^5^. The study cohort hence does not span periods with potentially different interpretations of the ASA classification system. Further studies of data before and after the 2020 ASA update could be done to evaluate the implications of ASA discordance in special populations, such as obstetric and pediatric patients.

The data used was derived from clinical databases, rather than administrative or financial records. Furthermore, neither surgeons nor anesthesiologists have financial incentives tied to ASA scoring at our institution. This eliminates an important source of bias as its presence has been shown to be associated with potential upcoding of the ASA class^32^.

A limitation of our study is that the assignment of ASA class by surgeons and anesthesiologists for each patient was not done simultaneously. At our institution, surgeons assign the ASA class when listing the patient for surgery and anesthesiologists assign their class after that at the preoperative assessment. As such, while the surgeon is completely blinded to the anesthesiologist’s class, the anesthesiologist could be aware of the surgeon’s class. However, our anesthesiologists generally make an independent assessment of the patient’s healthcare status. The anesthesiologist assessment is also closer to the day of surgery than the surgeon’s and hence the anesthesiologist’s class has better recency. It is also possible that the patient’s health could have deteriorated in the period of time between the surgeon and anesthesiologist review, accounting for class discordance and association with poorer outcomes. However, the waiting time for preoperative assessment at our institution is generally short and most elective surgeries are premised on a relatively stable patient physical status. We do not deem this to be a major source of bias.

As near- contemporaneous ASA scoring was mandatory for both anesthesiologist and surgeon during the study period, potential sources of bias (e.g. recall bias, selection bias) that may affect retrospective studies are much less likely in our study. There was a very small proportion of potential patients (264 patients, < 1%) who had missing anesthesiologist ASA class. However, as addressed in Supplementary Table S2, this is unlikely to be a major source of bias.

As our study only included patients who underwent elective surgery, its findings should not be generalized to emergency cases. Cardiothoracic, burns, and transplant surgery patients were also excluded, and our results may not apply in these groups of patients. Finally, as this was a single center study, this may limit generalizability, particularly in centers where ASA class impacts financial reimbursements (which is not present in our center) or centers with significantly different care patterns or patient comorbidity profiles.

### Opportunities for future work

Our study data did not contain information that could individually identify the anesthesiologists or the surgeons assigning ASA class. As such, we were unable to control for clinician factors that might have influenced the accuracy of the ASA classification, such as level of training and seniority. Our information about comorbidities assessed by the clinicians, which directly impacts the ASA class, was limited to the anesthesiologists only (as there was no standardized assessment form for surgeon-assessed comorbidities during the period of study). Future analyses of ASA discordance may investigate these aspects further, to better understand the mechanisms of ASA discordance and other possible factors that influence it.

The association of discordant ASA classification with adverse patient outcomes is a cause for concern. Besides further education and reinforcement of standard ASA examples, there may be a need for quality improvement studies to determine if specific conditions require more detailed or contextualized examples within the institution. Discordant ASA classes may be a red flag for missed comorbidities or interval development of new comorbidities, and mandatory cross-specialty review in ASA discordant cases is a potential intervention to ensure that patients are accurately assessed and appropriately prepared for surgery.

## Conclusion

In a large single-center cohort study that was performed after the 2014 update of the ASA class, there was moderate concordance between ASA classes assigned by anesthesiologists and surgeons in patients undergoing elective surgery. The majority of discordant patients were assigned a lower class by surgeons and is likely due to differences in recognition and grading of comorbidities. Patients with discordant ASA classes, and in particular those assigned lower ASA classes by surgeons, had a higher likelihood of 30-day mortality, 1-year mortality, and ICU admission > 24 h. Our results suggest a need for improvement in the standardization of ASA scoring and that discordant ASA assessments may be a red flag for missed comorbidities.

## Supplementary Information

Supplementary Tables.

## Data Availability

The datasets used and/or analyzed during the current study are available from the corresponding author on reasonable request.
