# Supplementary material for: Discordant American Society of Anesthesiologists Physical Status Classification between anesthesiologists and surgeons and its correlation with adverse patient outcomes
Source: Sci Rep. 2022 May 2;12:7110. doi: 10.1038/s41598-022-10736-5 (PMC9061797; doi:10.1038/s41598-022-10736-5)
Supplement: Supplementary file 1 — Supplementary Tables. [file 41598_2022_10736_MOESM1_ESM.pdf]

Discordant American Society of Anesthesiologists Physical Status Classification between Anesthesiologists and Surgeons and its correlation with Adverse Patient Outcomes

Charlene Xian Wen Kwa [1] (MMed), Jiaqian Cui [1] (MBChB), Daniel Yan Zheng Lim [2] (MBBS), Yilin Eileen Sim [1] (MMed), Yuhe Ke [1] (MBBS), *Hairil Rizal Abdullah [3] (MMed, MSc)

[1] Department of Anesthesiology, Singapore General Hospital, Outram Road, Singapore 169608

[2] Health Service Research Unit, Medical Board, Singapore General Hospital, Outram Road, Singapore 169608

[3] Duke-NUS Medical School, 8 College Road, Singapore 169857

charlene.kwa.x.w@singhealth.com.sg, jiaqian.cui@mohh.com.sg, daniel.lim@mohh.com.sg, eileen.sim.y.l@singhealth.com.sg, yuhe.ke@mohh.com.sg

*Corresponding author:

Hairil Rizal Abdullah MBBS MMed MSc

Senior Consultant, Department of Anesthesiology and Perioperative Medicine, Singapore General Hospital

Clinical Associate Professor, Duke-NUS Medical School

hairil.rizal.abdullah@singhealth.com.sg

Department of Anesthesiology, Singapore General Hospital, Outram Road, Singapore 169608

Telephone number: +65 63214220

Table S1: The 2014 ASA-Physical Status Classification System with approved examples

ASA-Physical Status Class	Definition	Examples, Including, but Not Limited to
**I**	A normal healthy patient	Healthy, nonsmoking, no or minimal alcohol use
**II**	A patient with mild systemic disease	Mild diseases only without substantive functional limitations. Examples include (but not limited to) current smoker, social alcohol drinker, pregnancy, obesity (30 < BMI < 40), well-controlled DM/HTN, mild lung disease
**III**	A patient with severe systemic disease	Substantive functional limitations; one or more moderate to severe diseases. Examples include (but not limited to) poorly controlled DM or HTN, COPD, morbid obesity (BMI ≥ 40), active hepatitis, alcohol dependence or abuse, implanted pacemaker, moderate reduction of ejection fraction, ESRD undergoing regularly scheduled dialysis, premature infant PCA < 60 weeks, history (> 3 months) of MI, CVA, TIA, or CAD/stents
**IV**	A patient with severe systemic disease that is a constant threat to life	Examples include (but not limited to) recent (< 3 months) MI, CVA, TIA, or CAD/stents, ongoing cardiac ischemia or severe valve dysfunction, severe reduction of ejection fraction, sepsis, DIC, ARDS, or ESRD not undergoing regularly scheduled dialysis
**V**	A moribund patient who is not expected to survive without the operation	Examples include (but not limited to) ruptured abdominal/thoracic aneurysm, massive trauma, intracranial bleed with mass effect, ischemic bowel in the face of significant cardiac pathology or multiple organ/system dysfunction
**VI**	A declared brain-dead patient whose organs are being removed for donor purposes	

The addition of "E" denoted emergency surgery: an emergency is defined as existing when delay in treatment of the patient would lead to a significant increase in the threat to life or body part.

ARDS = acute respiratory distress syndrome; BMI = body mass index; CAD = coronary artery disease; COPD = chronic obstructive pulmonary disease; CVA = cerebrovascular accident; DIC = disseminated intravascular coagulation; DM = diabetes mellitus; ESRD = end-stage renal disease; HTN = hypertension; MI = myocardial infarction; PCA = post conceptual age; TIA = transient ischemic attack.

Table S2: Table of differences between final cohort and excluded patients

		Final cohort [n=46284]	Excluded patients [n=264]	p-value[1]
**Male sex, No.(%)**		21474 (46.4)	136 (49.5)	0.39
**Age (Time of Surgery), mean (SD)**		58.0 (16.0)	59.0 (16.0)	0.27
**Race, No.(%)**	**Chinese**	34560 (74.7)	204 (74.1)	0.99
	**Indian**	4459 (9.6)	27 (9.8)	
	**Malay**	4111 (8.9)	25 (9.1)	
	**Others**	3154 (6.8)	19 (6.9)	
**Creatinine > 2mg/dl, No.(%)**		2281 (4.9)	13 (4.7)	0.81
**Diabetes mellitus on Insulin, No.(%)**		1750 (3.8)	2 (0.73)	<0.05
**History of Congestive heart failure, No.(%)**		1065 (2.3)	0 (0)	<0.05
**History of Cerebrovascular accident, No.(%)**		1529 (3.3)	5 (1.8)	0.22
**History of Ischemic heart disease, No.(%)**		4635 (10.0)	6 (2.2)	<0.05
**History of Hypertension, No.(%)**		19225 (41.5)	91 (33.1)	<0.05
**History of Smoking, No.(%)**		4327 (9.4)	10 (3.6)	<0.05
**Surgical Specialty, No.(%)**	**Orthopedics**	14523 (31.4)	85 (30.9)	0.25
	**General Surgery**	11294 (24.4)	72 (26.2)	
	**Urology**	6403 (13.8)	42 (15.3)	
	**Obstetrics and Gynecology**	4799 (10.4)	17 (6.2)	
	**Otorhinolaryngology**	2844 (6.1)	15 (5.5)	
	**Vascular**	2146 (4.6)	16 (5.8)	
	**Plastics**	1468 (3.2)	5 (1.8)	
	**Neurosurgery**	730 (1.6)	7 (2.6)	
	**Others**	2077 (4.5)	16 (5.8)	
**Death within, No.(%)**	**30 days**	212 (0.5)	3 (1.1)	0.32
	**1 year**	1661(3.6)	13 (4.7)	0.52
**Admitted to ICU >24 Hours, No.(%)**		834 (1.8)	2 (0.73)	0.18
**Total Stay in Days, mean (SD)**		6.0 (13.0)	8.0 (15.0)	0.51

[1] p-value for difference between final cohort and excluded patients

Excluded patients who had missing anesthesiologist ASA classes are represented here. There were no statistically significant differences between patients in the final cohort of our study and excluded patients for demographic variables (age, sex, race) and clinical outcomes. Fewer of the excluded patients had anesthesiologist-assessed comorbidities, and the differences were statistically significant for some.

Table S3: Effect of Discordant ASA Class on Clinical Outcomes

	Death within 30 days			Death within 1 year			Admitted to ICU >24 Hours		
	**No. (%)**	**Odds ratio (95% confidence interval)**	**p-value**	**No. (%)**	**Odds ratio (95% confidence interval)**	**p-value**	**No. (%)**	**Odds ratio (95% confidence interval)**	**p-value**
**Overall [n=46284]**	212 (0.5)	-	-	1661(3.6)	-	-	834 (1.8)	-	-
**Concordant ASA Class [n=31186]**	108 (0.4)	-	-	961 (3.1)	-	-	461 (1.5)	-	-
**Discordant ASA Class [n=15098]**	104 (0.7)	2.00 (1.52-2.62)	<0.0001	700 (4.6)	1.53 (1.38-1.69)	<0.0001	373 (2.5)	1.69 (1.47-1.94)	<0.0001
**Concordant ASA Class [n=31186]**	108 (0.4)	-	-	961 (3.1)	-	-	461 (1.5)	-	-
**Surgeon ASA class lower [n=11985]**	92 (0.8)	2.23 (1.68-2.94)	<0.0001	621 (5.2)	1.72 (1.55-1.90)	<0.0001	284 (2.4)	1.61 (1.39-1.87)	<0.0001
**Anesthesiologist ASA class lower [n=3113]**	12 (0.4)	1.11 (0.58-1.94)	0.724	79 (2.5)	0.82 (0.64-1.03)	0.092	89 (2.9)	1.96 (1.55-2.45)	<0.0001
**Concordant ASA Class [n=31186]**	108 (0.4)	-	-	961 (3.1)	-	-	461 (1.5)	-	-
**Surgeon ASA class lower by 2-3 levels* [n=372]**	7 (1.9)	5.52 (2.31-11.09)	<0.0001	32 (8.6)	2.96 (2.01-4.21)	<0.0001	19 (5.1)	3.59 (2.17-5.58)	<0.0001
**Surgeon ASA class lower by 1 level [n=11613]**	85 (0.7)	2.12 (1.59-2.81)	<0.0001	589 (5.1)	1.68 (1.51-1.87)	<0.0001	265 (2.3)	1.56 (1.33-1.81)	<0.0001
**Anesthesiologist ASA class lower by 1-2 levels* [n=3113]**	12 (0.4)	1.11 (0.58-1.94)	0.724	79 (2.5)	0.82 (0.64-1.03)	0.092	89 (2.9)	1.96 (1.55-2.46)	<0.0001

* Categories collapsed due to small numbers

Evaluation of clinical outcomes in association with discordant ASA classes. Further stratification of discordant ASA classes here showed that a lower surgeon ASA class was associated with all three categories of adverse clinical outcomes. The lower the surgeon ASA class was compared to the anesthesiologist class, the higher the risk for all three adverse clinical outcomes. Comparatively, a lower anesthesiologist ASA class was only associated with ICU admission >24 hours but not with death within 30 days or 1 year.

Table S4: Subgroup analysis on the effect of discordant ASA class on clinical outcomes within ASA class 1-2 groups, and ASA class 3-4 groups

		Death within 30 days		Death within 1 year		Admitted to ICU >24 Hours	
**Surgeon ASA class**	**Anesthesiologist ASA class**	**Odds ratio (95% confidence interval)**	**p-value**	**Odds ratio (95% confidence interval)**	**p-value**	**Odds ratio (95% confidence interval)**	**p-value**
1	2	$2.16e^{7}$ ($3.09e^{-150}$-NA)	0.993	$2.64e^{7}$ ($1.43e^{-8}$ - $6.27e^{104}$)	0.982	3.94 (1.24-14.78)	0.025
2	1	$9.31e^{6}$ ($1.33e^{-150}$-NA)	0.994	$4.05e^{7}$ ($2.20e^{-8}$ - $9.63e^{104}$)	0.981	4.46 (1.70-15.30)	0.006
3	4	5.24 (1.97-11.60)	<0.001	1.24 (0.63-2.24)	0.501	11.60 (7.12-18.71)	<0.001
4	3	5.57 (3.27 -9.11)	<0.001	2.54 (1.90-3.39)	<0.001	6.37 (4.72-8.54)	<0.001

A subgroup analysis on the effects of discordant ASA class on clinical outcomes was done within the lower ASA class 1-2 groups, as well as higher ASA class 3-4 groups. Within the ASA class 1-2 groups, the reference group was ASA class 1 rated by both anesthesiologist and surgeon. Within the ASA class 3-4 group, the reference group was ASA class 3 rated by both anesthesiologist and surgeon. When there was discordance between ASA class 1-2, there was no significant correlation with any of the 3 clinical outcomes (death within 30 days, 1 year, and admission to ICU >24 hours). In contrast, when there was discordance between ASA class 3-4, all combinations of ratings were significant for the outcomes of death at 30 days and ICU admission >24h, and for death at 1 year when surgeon ASA class was lower.
